# Identification of GSK3 Family Genes in Pear and Their Expression Analysis Under Drought Stress

**DOI:** 10.3390/life15030349

**Published:** 2025-02-24

**Authors:** Kairan Hu, Ziyi Zhang, Guoliang Li, Shuliang Zhao, Yali Zhang, Qingjiang Wang, Fuhou Cheng

**Affiliations:** 1 School of Landscape and Ecological Engineering, Hebei University of Engineering, Handan 056038, China; 18833877839@163.com (K.H.);; 2 Institute of Biotechnology and Food Science, Hebei Academy of Agriculture and Forestry/Key Laboratory of Plant Transgenic Centre of Hebei Province, Shijiazhuang 050051, China; 3 Handan City Garden Bureau, Handan 056000, China

**Keywords:** pear, GSK3, genome-wide, expression pattern

## Abstract

Members of the glycogen synthase kinase 3 (GSK3) family in plants, as a class of serine/threonine protein kinases, have been demonstrated to play crucial roles in a wide range of biological processes and environmental stresses. However, the GSK3 gene family has not been analyzed in pears. In this study, 12 GSK3 gene family members were identified in the *Pyrus bretschneideri* genome. These genes were located on 10 chromosomes and phylogenetically classified into four subfamilies. All 12 PbGSK3 proteins possessed highly conserved domains. The results demonstrated the structural characteristics of all 12 *PbGSK3s* and the evolutionary processes of their putative proteins. The presence of several cis-acting elements in the promoter region of the *PbGSK3s* associated with hormonal and stress responses suggested that the *PbGSK3s* might be involved in the growth and development of pears and in stress response. The expression profiles of *PbGSK3s* under drought stress were also analyzed. When the pears were subjected to drought stress for different durations, the expression patterns of 12 *PbGSK3s* exhibited variations. The findings would provide a scientific foundation for further exploration of the potential functions of the GSK3 genes in pears.

## 1. Introduction

Due to the specific ability to phosphorylate glycogen synthase, glycogen synthase kinase (GSK) has attracted significant attention from the scientific community. Notably, GSK3, as a non-receptor serine/threonine protein kinase, has been shown to exhibit distinct functions and play an important role in cell signal transduction and metabolic regulation [1]. In animals, it has been confirmed that GSK3 proteins participate in signal transduction for various life activities, including glycogen synthesis, insulin regulation, transcriptional activation of multiple proteins, and developmental regulation [2]. In plants, the GSK3 gene family also plays a crucial role in physiological processes such as osmotic stress response, wound response, and Oleandrin lactone signaling [3,4,5]. The first GSK3 gene in the plant was identified in *Medicago sativa* L., followed by the discovery of 10 and 9 members in *Arabidopsis thaliana* L. and *Oryza sativa* L., respectively. The GSK3 genes were classified into four subfamilies, and it is noteworthy that their homologous genes are also classified into the same number of subfamilies [6,7,8]. Subsequently, GSK3 members have been identified in many plants, such as *Triticum aestivum* L., *Nicotiana tabacum* L., and *Brassica napus* L. [9,10].

GSK3 protein kinases have been shown to play extensive and crucial roles in plant growth and development, abiotic stress response, and pathogen defense. During plant growth and development, GSK3 proteins such as BIN2/AtSK21 phosphorylate multiple substrates, including EGL3, TTG1, ARF7, and ARF19, thereby participating in the processes of root hair formation, lateral root development, and xylem and phloem differentiation [11,12,13,14,15]. These processes are essential for the establishment of plant root structure and function. In plant response to stresses, multiple GSK3 members, AtSK11, AtSK22, AtSK13, AtSK31, and AtSK32, have been observed to exhibit increased transcription levels or enhanced activity under salt stress conditions. By activating downstream targets such as G6PD, these genes contribute to enhancing plant salt tolerance [11,12,13,14,15]. Notably, the increased expression of AtSK11 resulted in the improvement of plant salt resistance [16]. BIN2 can phosphorylate transcription factors RD26 and TINY and then activate dehydration-responsive genes and positively regulate the plant drought stress response [17]. AtSK21/BIN2 has been identified as a key factor in Oleuropein lactone (BR) and ABA signaling pathway [18]. It has been demonstrated that BIN2 phosphorylates SnRK2.2 and SnRK2.3, enhancing their ability to phosphorylate downstream substrate ABA-responsive element-binding factors (ABFs), thus positively regulating the ABA signaling pathway and affecting plant growth, development, and stress response [18]. Plant GSK3 protein kinases are also involved in the pathogen response process regulated by the MAP kinase pathway. For instance, the *Medicago sativa* GSK3 protein kinase MsK1 has been shown to enhance plant susceptibility to pathogens, thereby suggesting a potential negative regulatory role for MsK1 in plant immune responses [19].

The GSK3 family has been identified, and its functions have been analyzed in multiple species. However, it has not yet been systematically characterized in pears. This study utilized bioinformatics to screen for GSK3 family genes, conducting classification, physicochemical properties, conserved domain, and putative protein structures in pears. A phylogenetic tree was constructed with GSK3 family genes from *Pyrus bretschneideri*, *Arabidopsis thaliana*, and *Oryza sativa* to analyze the evolutionary relationships. Furthermore, the expression patterns of GSK3 genes under drought stress were investigated in pears. This study provides a theoretical basis for further research on the roles of pear GSK3 genes in drought stress.

## 2. Materials and Methods

### 2.1. Identification of the GSK3 Gene Family in Pears

The genome sequences of ‘Dangshan Suli’ (*P. bretschneideri*) V1.1 were downloaded from the Genome Database for Rosaceae (GDR) (http://www.rosaceae.org/ accessed on 16 August 2024). The GSK3 Pfam (PF00069) was obtained from the Pfam database (http://pfam.xfam.org/ accessed on 16 August 2024). The hidden Markov model (HMM) profiles were used to search against the pear protein sequence data using default parameters and removed the non-representative transcripts. Then, the remaining sequences were checked for the conserved GSK3 domain using SMART (http://smart.embl-heidelberg.de/ accessed on 16 August 2024).

### 2.2. Phylogenetic Tree Construction

The PbGSK3 protein sequence was constructed by Neighbor Joining (NJ) using MEGA (V6.06) software with the Arabidopsis GSK3 protein sequences downloaded from the TAIR (https://www.arabidopsis.org/ accessed on 19 August 2024) and the Rice GSK3 protein sequences downloaded from the database (http://rice.uga.edu/ accessed on 19 August 2024). The phylogenetic tree was then refined using the iTOL platform (https://itol.embl.de/ accessed on 19 August 2024).

### 2.3. Synteny Analysis

The genome sequences of ‘Dangshan Suli’ were analyzed by the One Step MCScan plug-in in TBtools V2.152 [20]. The resulting data file (.collinearity), the new annotation file (.gff), and the original control file (.ctl) were then processed to remove the unassembled scaffolds to the chromosomes. The results of the synteny analysis were visualized using the visualization tool Circle Gene View in TBtools [20].

### 2.4. Protein Characterization and Sequences Analyses

The length of amino acids, molecular weight, and isoelectric point (pI) were analyzed with PbGSK3 sequences in ExPASy (http://web.expasy.org/protparam/ accessed on 18 August 2024). Subsequently, the genome and CDS sequences of the pear GSK3 family were uploaded to the GSDS 2.0 website (http://gsds.cbi.pku.edu.cn/ accessed on 17 August 2024) in order to analyze gene structure. The MEME website (https://meme-suite.org/meme/ accessed on 19 August 2024) was then used to investigate the conserved motifs of the pear GSK3 protein sequence. The MEME website (https://meme-suite.org/meme/ accessed on 19 August 2024) was then used to analyze the conserved motifs of the pear GSK3 gene family members, with the number of motifs set to 10 and other parameters left at their default values. The results were then visualized and mapped using TBtools [20]. The Batch SMART function in TBtools was then used to analyze the functional domains of the pear GSK3 family member protein sequences obtained and to visualize these for mapping [20].

### 2.5. Secondary and Tertiary Structure Analysis of the PbGSK3s

The screened pear GSK3 family protein sequences were subsequently subjected to secondary structure prediction using Prabi tool (https://npsa-prabi.ibcp.fr/cgi-bin/npsa_automat.pl?page=/NPSA/npsa_sopma.html/ accessed on 19 August 2024). In addition, the tertiary structures of pear proteins were modeled and displayed by the Swiss-Model interactive tool (https://swissmodel.expasy.org/interactive/ accessed on 19 August 2024).

### 2.6. Promoter Cis-Acting Elements Analysis

The sequence of 2000 base pairs upstream of the PbGSK3 family gene was selected from the sweet potato genome data, and the sequence of 2000 base pairs upstream of the PbGSK3 family gene was selected from the pear genome data by using the online website PlantCARE (https://bioinformatics.psb.ugent.be/webtools/plantcare/html/ accessed on 19 August 2024) to analyze cis-acting elements.

### 2.7. Expression Analysis of PbGSK3 Genes

Transcriptome data of drought-treated samples were downloaded from the NCBI SRA database under the accession numbers of SRR7193568- SRR7193572 (Bioproject PRJNA472429). Gene expression heat maps were then generated based on the FPKM values of the genes using TBtools [20].

## 3. Results

### 3.1. Identification and Phylogenetic Analysis of Pear GSK3 Genes

**Figure 1:**
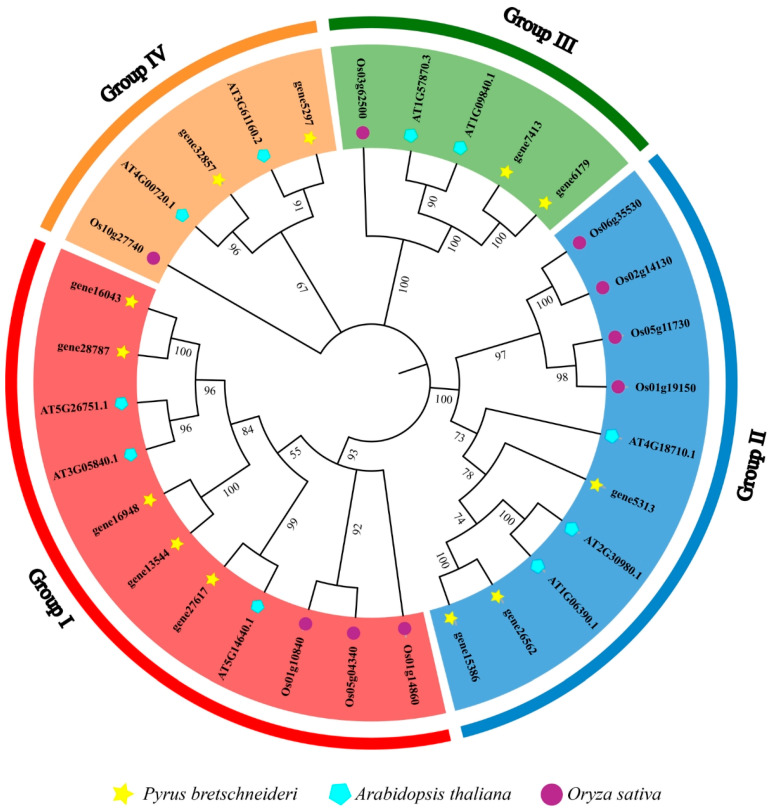
Phylogenetic analysis of the GSK3 proteins from different species.

Based on a genome-wide analysis, 12 *PbGSK3* genes (*gene6179*, *gene7413*, *gene5313*, *gene5297*, *gene15386*, *gene26562*, *gene27617*, *gene16948*, *gene13544*, *gene16043*, *gene28787*, and *gene32857*) were identified in pears. In order to elucidate the phylogenetic relationship of the PbGSK3s, a phylogenetic tree was constructed using 10 GSK3 protein sequences from A. thaliana and 9 and 12 GSK3 protein sequences from *O. sativa* L. and *P. bretschneideri* L. (Figure 1). The results revealed that the GSK3s in three species were classified into four subgroups. The distribution of GSK3 members across each subgroup subfamily was found to be uneven. Subgroup I contained five PbGSK3 members (gene16043, gene28787, gene16948, gene13544, gene276170), while subclades II (gene7413, gene6179) and IV (gene6643, gene41806) contained two PbGSK3 members each. Subclade III contained three PbGSK3 family members.

### 3.2. Synteny Analysis and Physicochemical Properties

**Figure 2:**
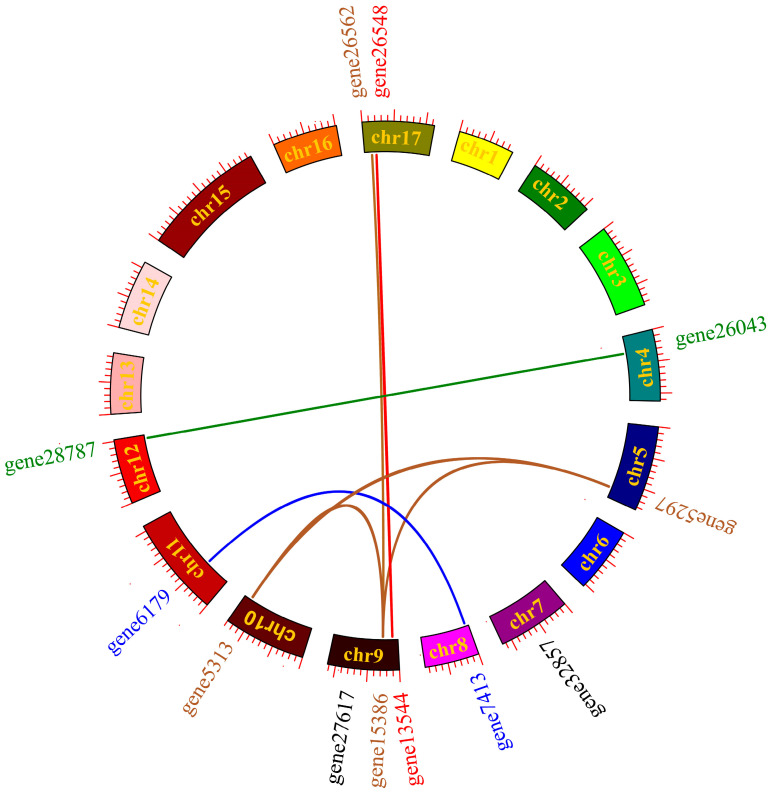
Synteny analysis of PbGSK3 genes.

The collinearity analysis of the 12 PbGSK3s revealed that they were unevenly distributed across 10 chromosomes (Figure 2). Specifically, gene5297 and gene15386 are co-located on chromosome 5, while gene26562 and gene16948 are co-located on chromosome 9. The analysis revealed a collinearity relationship among the PbGSK3s, with 10 out of the 12 PbGSK3s exhibiting collinearity. A total of seven gene pairs were identified, with certain PbGSK3s, such as gene15386, demonstrating collinearity with multiple genes, including gene5313, gene26562, and gene5297.

Detailed information on the *PbGSK3* genes is given in Table 1. The result demonstrated that the number of amino acids encoded by all 12 GSK3 genes ranged from 378aa to 474aa, with protein molecular weights ranging from 42,763.54 Da to 53,517.11 Da. Furthermore, the isoelectric points of all 12 GSK3 proteins were found to be greater than 7 in pears.

### 3.3. Gene Structure and Conserved Domains

**Figure 3:**
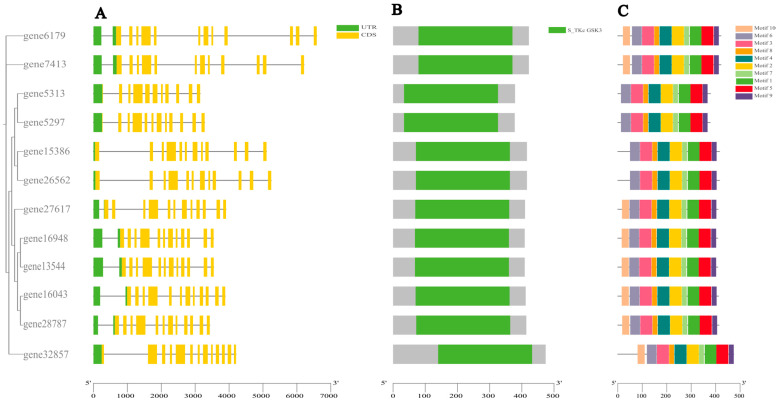
Gene structure, protein conserved domain, and conserved motif of GSK3 gene family in pears. (**A**) Exon and intron structure of the PbGSK3s; (**B**) Protein conserved structural domains; (**C**) Conserved motifs.

The results showed that the number of PbGSK3 exons ranged from 11 to 13, with slight variations in genomic locus length. Among them, gene6179 possessed the longest genomic locus (Figure 3). PbGSK3 structure from the same clade exhibited similarities, while gene structure from different clades showed differences, possibly due to distinct functions performed by different subfamilies. The online software MEME was utilized to detect 10 conserved motifs (motif1-motif10) in the 12 PbGSK3s. The results revealed that each GSK3 contained 9 to 10 motifs distributed within 500 amino acids. Motifs 1 to 9 were common to all members, and the arrangement order of the motifs was also identical. Among these GSK3 genes, gene5313, gene5297, gene15386, and gene26562 contained nine motifs. Studies have shown that motifs 1 to 9 represent a complete GSK3 domain, with motif6 located at the 5′ end and motif9 at the 3′ end of the domain.

### 3.4. Secondary and Tertiary Structures of GSK3 Proteins

Secondary structures of GSK3s were of significant importance in the transmission of genetic information, the structural similarity among protein members, and the potential mechanisms of protein expression and regulation (Table 2). The secondary structures of the GSK3 family were primarily composed of α-helices (33.5–40.08%) and random coils (32.77–39.42%), while β-turns (8.73–13.2%) and extended strands (14.35–20.15%) account for a smaller proportion. The structural similarity among PbGSK3s was often closely related to their functional relevance.

**Figure 4:**
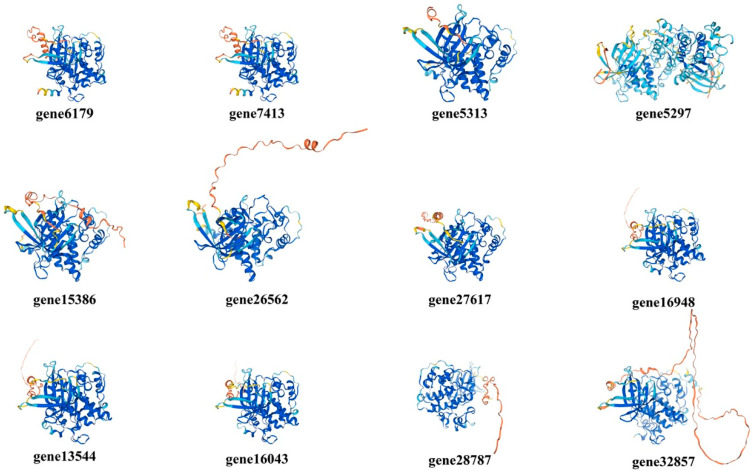
Protein tertiary structure predictions of PbGSK3.

The tertiary structure of 12 PbGSK3s was predicted using the online tool Swiss-Model (Figure 4). The results demonstrated that the tertiary structures were similar in proportion to their secondary structures, with only slight differences in spatial conformation. The structural similarity might be attributable to the common ancestry of the protein members, while the structural differences might be attributable to variations in gene expression time, spatial location, or level.

### 3.5. Analysis of Cis-Acting Elements in the Promoter of PbGSK3s

**Figure 5:**
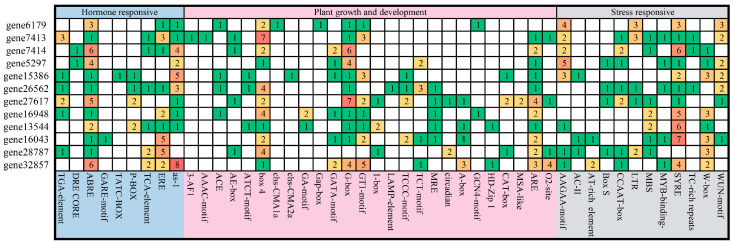
Analyses of cis-acting elements in the PbGSK3 gene promoters.

The cis-acting elements in the promoter sequences of PbGSK3s were predicted in PlantCARE. All cis-regulatory elements in the GSK3 gene promoter region were classified into three categories: hormone response, plant growth and development, and stress response (Figure 5). Among the hormone response elements, the ABRE response element was found to be the most abundant, with 32 instances across 12 promoters of *PbGSK3*. BOX 4 and G-BOX motifs were the most prevalent related to plant growth and development, each found in 28 instances, and the stress-related element STRE was the most numerous, with 32 instances in 11 of the 12 *PbGSK3s*.

Given the function of GSK3s in plant response to drought, a detailed investigation was conducted of the cis-acting elements involved in drought stress response. All gene promoters contained ABRE, which played a crucial role in drought response. The promoters of *gene7413* and *gene32857* contained the highest number of ABREs. It indicated that the promoters of these two genes contained more regulatory sites related to drought stress. In addition, *gene7413*, *gene5297*, *gene26562*, *gene16043*, and *gene32857* contained MYB drought-responsive elements.

Additionally, *gene6179*, *gene7413*, *gene26562*, *gene27617*, and *gene28787* all contained LTR low-temperature responsive elements.

The results of the cis-element analyses indicated that PbGSK3s might play important roles in a wide range of plant developmental processes and plant responses to various abiotic and biotic stresses.

### 3.6. Expression Pattern of PbGSK3s Response to Drought

**Figure 6:**
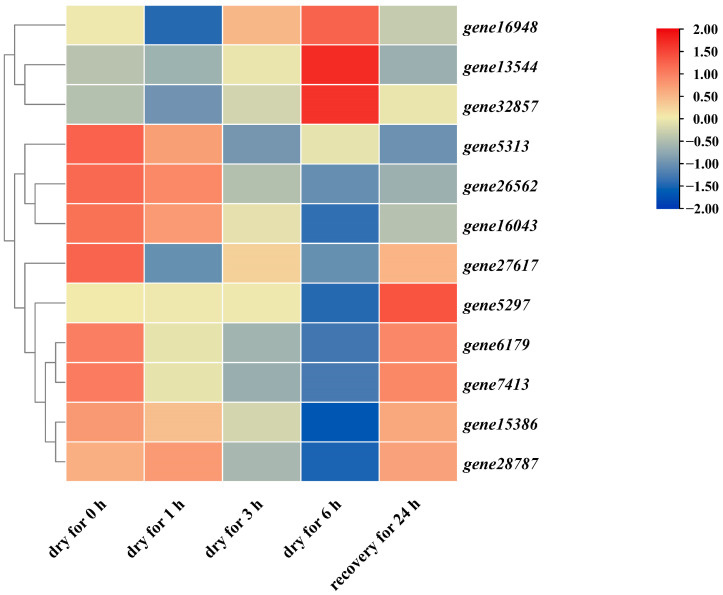
Transcriptional profiling and expression levels of *PbGSK3s* response to drought.

In order to explore the expression change of PbGSK3s under drought stress, a heat map was made using published transcriptome data (Figure 6). The results showed that the expression levels of all 12 *PbGSK3s* exhibited varying degrees after exposure to drought stress. Specifically, *gene16948*, *gene13544*, and *gene32857* exhibited a slight decrease in expression one hour after drought stress, followed by a significant increase over time, peaking at six hours and then decreasing to their initial levels twenty-four hours after rewatering. And *gene5313*, *gene26562*, *gene16043*, and *gene27617* all demonstrated a gradual decline in expression following drought stress, reaching their lowest at six hours and subsequently increasing at twenty-four hours after rewatering, with *gene27617* exhibiting the most substantial increase. The expression level of *gene5297* exhibited no significant change from 0 to 3 h after drought stress but decreased at 6 h. Following rehydration, a significant increase in expression was observed after 24 h. The expression amount of *gene6179*, *gene7413*, *gene15386*, and *gene28787* declined from 0 to 6 h following drought stress and then increased after rehydration. The divergent expression patterns of *PbGSK3* in pear response to drought stress represent the functional diversity and distinctions among the family members, thereby implicating their different functions when pears suffer environmental stresses.

## 4. Discussion

The number of GSKS genes in each species was different. Twenty-two GSK3 genes were identified in the wheat genome [21]. Nine GSK3 genes were identified in the potato genome [22]. Twenty-five GSK3 gene family members were identified in the soybean genome [23]. This research showed that GSK3 was known to be vital in plant growth, development, and response to environmental stresses in plant species. However, the role of GSK3 in pears has been scarcely studied. In this study, 12 members of the GSK3 family were identified in pear. They are located on 10 chromosomes (4, 5, 7, 8, 9, 10, 11, 12, 17). All 12 genes possessed the S_TKc domain, indicating the conservation of the GSK3 gene in evolutionary relationships [24]. All 12 PbGSK3s can be clarified into four subfamilies by phylogenetic tree analysis. The number of GSK3 subfamilies is the same as that of Arabidopsis and rice [25,26]. Furthermore, the analysis revealed that the genetic relationship between pear and Arabidopsis was more closely related than that of pear and rice. This outcome can be attributed primarily to the fact that both pear and Arabidopsis are classified as dicotyledonous plants, whereas rice is a monocotyledonous plant [27]. The identification of seven gene pairs with gene duplication events (seven segmental duplication gene pairs and zero tandem duplication gene pairs) was based on the findings of the collinearity analysis. It indicated that these genes in the genome experienced segmental duplication.

An analysis of the physicochemical properties of the 12 PbGSK3 family member proteins was conducted, which revealed that the isoelectric points of all 12 of its PbGSK3 members were greater than 7. This finding indicates that pear GSK3 is a basic protein. The prediction of the high-level protein structures of PbGSK3 members demonstrated that the results of the tertiary structure were largely consistent with the secondary structure, thereby indicating that the protein characteristics of the 12 GSK3 gene family members were homogeneous in pears.

A number of cis-acting elements associated with growth, stresses, and development have been identified within the promoters of *PbGSK3s*. The diversity and abundance of these elements are indicative of their involvement in various aspects of plant growth and stress regulation. Our analysis has revealed the presence of multiple response elements within the promoter regions of PbGSK3s, including ABRE, MYB, MBS, LTR, W-box, DRE, TGA-element, etc. These elements have been demonstrated to participate in the expression of stress-related genes in plants by binding to specific transcription factors. In the context of drought stress, the expression levels of genes 13544 and 32857, which contain ABRE and MYB drought stress response elements, respectively, have been observed to increase in response to drought stress. This finding is consistent with the results of previous research on the drought stress response of the GSK3 gene family in celery [28]. This finding suggests that the GSK3 gene family may respond to stress by activating hormone signals under adverse conditions, thereby playing a defensive and protective role in plant growth and development.

The expression level of PbGSK3s revealed that the expression level of genes 16948, 13544, and 32857 were all elevated after exposure to drought stress. These findings are consistent with other studies, which found that one of GSK3, AtSK21 (BIN2), is involved in the plant’s drought stress response and interacts with WRKY54 through phosphorylation to enhance drought resistance [29,30]. BIN2, a member of GSK3, interacts with RD26 (Responsive to Desiccation 26) and promotes its phosphorylation, enhancing the stability and transcriptional activation ability of RD26, thereby improving the plant’s drought tolerance. This finding confirms the important role of GSK3 proteins in the plant’s drought resistance mechanism. Meanwhile, there is a crosstalk between AtSK21 and AtSK22 in plant development, drought, and salt stress signaling. Specifically, the phosphorylation of RD26 by BIN2 not only stabilizes RD26 but also prompts it to activate a series of dehydration-responsive genes, driving the plant’s response to drought stress. Collectively, these studies illuminate the intricate regulatory network of GSK3 proteins in plant response to environmental stresses [17,31].

## 5. Conclusions

A comprehensive genome-wide analysis of PbGSK3 revealed the identification of 12 PbGSK3s, accompanied by the observation of highly conserved key motifs regulating GSK3 kinase activity. It is postulated that the PbGSK3s play a pivotal role in the processes of pear growth and development, as well as in the context of adverse stress conditions. This provides a scientific foundation for the subsequent investigation of the gene functions of the PbGSK3 family. However, the biological functions of some of the genes in this family have yet to be verified, and the functions and roles of some specific genes in this family can be verified in depth in the future, which will be conducive to the cultivation of excellent stress-resistant varieties of pear.

## Figures and Tables

**Table 1:** Physicochemical properties of PbGSK3 proteins.

Name	Chromosome Location	Exon Number	Protein Length (aa)	Molecular Weight (Da)	PI	CDs Length (bp)
gene6179	11	12	422	47,803.93	8.37	1269
gene7413	8	12	422	47,886.05	8.19	1269
gene5313	10	11	379	42,928.64	8.62	1140
gene5297	5	12	378	42,763.54	8.62	1137
gene15386	5	12	416	47,167.49	8.73	1251
gene26562	17	12	416	47,122.46	8.73	1251
gene27617	9	12	410	46,042.42	8.95	1233
gene16948	17	12	409	46,127.49	8.81	1230
gene13544	9	12	409	46,229.58	8.7	1230
gene16043	4	12	412	46,806.17	8.7	1239
gene28787	12	12	414	46,821.24	8.55	1245
gene32857	7	13	474	53,517.11	7.31	1425

**Table 2:** PbGSK3 protein secondary structure.

Name	Alpha Helix	Beta Turn	Extended Strand	Random coil
gene6179	38.63	10.19	17.77	33.41
gene7413	37.2	10.43	18.48	33.89
gene5313	35.88	10.03	16.09	37.99
gene5297	36.77	8.73	16.14	38.36
gene15386	34.86	9.13	16.59	39.42
gene26562	34.62	9.86	16.11	39.42
gene27617	36.83	9.76	19.76	33.66
gene16948	33.5	13.2	19.8	33.5
gene13544	34.47	12.22	19.07	34.23
gene16043	35.92	11.17	20.15	32.77
gene28787	36.71	10.63	19.32	33.33
gene32857	40.08	9.92	14.35	35.65

## Data Availability

Publicly available datasets were analyzed in this study. This data can be found here: [accession number SRR7193568-SRR7193572].
